# The Maintained Glycemic Target Goal and Renal Function Are Associated with Cardiovascular and Renal Outcomes in Diabetic Patients Following Stent-Supported Angioplasty for Renovascular Atherosclerotic Disease

**DOI:** 10.3390/jpm12040537

**Published:** 2022-03-28

**Authors:** Rafał Badacz, Anna Kabłak-Ziembicka, Agnieszka Rosławiecka, Daniel Rzeźnik, Jakub Baran, Mariusz Trystuła, Jacek Legutko, Tadeusz Przewłocki

**Affiliations:** 1 Department of Interventional Cardiology, Institute of Cardiology, Medical College, Jagiellonian University, 31-008 Krakow, Poland; rbadacz@gmail.com (R.B.); rzeznikd@poczta.onet.pl (D.R.); jakub_baran@yahoo.pl (J.B.); jacek.legutko@uj.edu.pl (J.L.); 2 Department of Interventional Cardiology, The John Paul II Hospital, 31-202 Krakow, Poland; agnieszkaroslawiecka@interia.pl (A.R.); tadeuszprzewlocki@op.pl (T.P.); 3 Noninvasive Cardiovascular Laboratory, The John Paul II Hospital, 31-202 Krakow, Poland; 4 Department of Vascular and Endovascular Surgery, John Paul II Hospital, 31-202 Krakow, Poland; m.trystula@szpitaljp2.krakow.pl; 5 Department of Cardiac and Vascular Diseases, Institute of Cardiology, Medical College, Jagiellonian University, 31-008 Krakow, Poland

**Keywords:** arterial occlusive disease, blood pressure, cardiovascular and renal outcomes, chronic kidney disease, endovascular procedures, renal artery, stents, target glycemic goals, type 2 diabetes mellitus

## Abstract

Patients with type 2 diabetes mellitus (T2DM) constitute a large proportion of patients with atherosclerotic renal artery stenosis (ARAS). However, the mechanism of impaired renal function and hypertension in this subset of patients is multifactorial. We aimed to investigate whether, in diabetic patients, renal function (RF), systolic (SBP) and diastolic blood pressure (DBP) values following stent-supported angioplasty (PTA) for ARAS have an impact on cardiovascular and renal outcomes. Methods: The study group included 93 patients with T2DM and resistant hypertension who underwent PTA for ARAS. The pre- and post-procedure (6 to 12, and 24 months) values of SBP, DBP, eGFR and glycaemia were obtained. The prospective follow-up of median 44 months was performed for combined outcome: major cardiac and cerebral events (MACCE) and progression to renal replacement therapy (RRT). Results: MACCE-RRT occurred in 46 (49.5%) patients, with higher incidence in patients with higher values of SBP (147.8 ± 25.8 vs. 136.7 ± 15.8 mmHg, *p* = 0.006), DBP (80.8 ± 13.3 vs. 74.4 ± 12.3 mmHg, *p* = 0.009), chronic kidney disease in stages 3B to 5 (*p* = 0.029) and those who have not obtained target glycemic goals compared to well-maintained T2DM (*p* = 0.007) at 24-months. On multivariate Cox analysis, well-maintained T2DM targets [Hazard Ratio (HR):0.27; 95% Confidence Interval (CI):0.13–0.57; *p* < 0.001], eGFR below 45 mL/min/m^2^ (HR: 2.20; 95%CI: 1.20–4.04; *p* = 0.011), previous stroke (HR:2.52; 95%CI:1.19–5.34; *p* = 0.015) retained their associations with MACCE-RRT, while BP values were not associated with the outcome. Conclusions: The post-procedural RF, maintained glycemic target goal and previous stroke are vital for the outcome in patients undergoing PTA for renovascular disease in diabetic patients.

## 1. Introduction

Patients with type 2 diabetes mellitus (T2DM) constitute a large proportion of patients with atherosclerotic renal artery stenosis (ARAS) [1]. Diabetes predisposes to renal vasculopathy, the incidence of which is probably underestimated in clinical settings [2,3]. As evidenced in autopsy studies, ARAS accounts for 10.1% incidence rate in diabetic patients with arterial hypertension, compared to 1–2% of hypertensive patients free from diabetes [2]. Consistently, among 545 patients with coronary artery disease (CAD), the incidence of T2DM was significantly increased (from 24.4% to 50%) in patients who also had multiple arterial diseases (carotid, renal or lower extremity) [3].

The mechanisms of impaired renal function (RF) and hypertension in diabetic patients are multifactorial and obviously not solely attributed to the incidence of ARAS [4]. However, ARAS leading to chronic renal ischemia and the activation of the renin–angiotensin–aldosterone axis exaggerates already existing diabetic nephropathy and blood pressure (BP) values [5]. Diabetic patients with ARAS are particularly exposed to major cardiac and cerebral events (MACCE) and renal replacement therapy (RRT) [6,7].

Alas, there is much uncertainty whether stent-supported angioplasty (PTA) of stenotic renal artery can improve RF, BP control and cardiovascular outcomes [8,9,10]. The data gathered in this field are ambiguous. 

In the present study, we aimed to investigate, in diabetic patients, whether RF, glycemic control, systolic (SBP) and diastolic blood pressure (DBP) values following PTA for ARAS have an impact on cardiovascular outcomes. In addition, we searched for biochemical, clinical and renal ultrasonographic (DUS) parameters that may be helpful for the prediction of procedure outcomes. 

## 2. Materials and Methods

The study group included 93 patients with T2DM who underwent PTA for ARAS in accordance with the guidelines [11,12], such as accelerated or refractory hypertension on at least 3 antihypertensive medications (including at least one diuretic), and /or RF impairment. The other inclusion criteria were pulmonary flash edema in the presence of a preserved systolic left ventricle ejection fraction (LVEF > 50%), exertional angina that could not be explained by the status of coronary arteries, bilateral ARAS or ARAS of the single functional kidney [11,12]. Exclusion criteria were a non-atherosclerotic etiology of ARAS, non-diagnostic renal DUS, lack of patient’s informed consent to participate in the study. The treatment period of T2DM, type of the treatment (insulin, oral medications), the efficacy of T2DM control were evaluated in all patients.

The distribution of traditional cardiovascular risk factors (hyperlipidemia, arterial hypertension, former or active smoking), as well as known coronary artery disease (CAD), history of myocardial infarction (MI), and stroke were recorded. Definitions of the above were adopted from the scientific statements of the European Society of Cardiology [13,14,15]. All patients obtained peri- and post-procedural optimal medical treatment according to recommendations of the respective societies [13,14,15].

The pre-procedure assessment of SBP and DBP, RF (serum creatinine and the estimated glomerular filtration rate; eGFR), the incidence of traditional cardiovascular risk factors and renal DUS were obtained in all patients.

The study complies with the Declaration of Helsinki and was approved by the Jagiellonian University Ethics Committee (KBET/392/B/2003; with further extensions). All participants signed a written informed consent.

**Figure 1:**
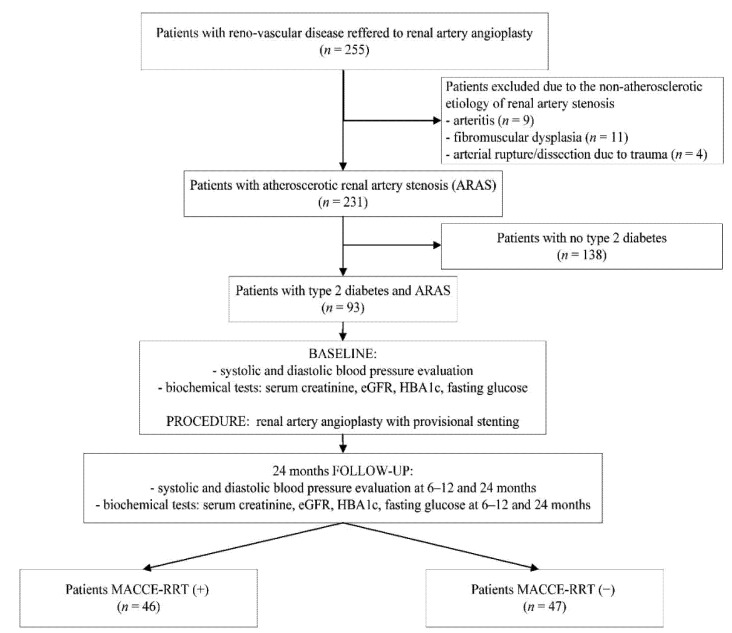
Study flowchart.

The study flowchart with overall study design is shown in Figure 1.

### 2.1. Blood Pressure and Renal Function Assessment

BP was measured according to the recommendations of the Joint National Committee on Prevention, Detection, Evaluation, and Treatment of High Blood Pressure VII [15,16]. Arterial hypertension was defined as SBP ≥ 140 and/or DBP ≥ 90 mmHg, respectively. Three to five measurements were carried out in the sitting position after 5-min resting period with a validated automatic oscillometric device, choosing a cuff based on arm circumference. The reported value was the average of the measurements. Office BP was recorded according to the 2018 ESH/ESC guidelines [15]. During clinic evaluation, staff recorded office BP in the same manner, moreover patients were asked to report the mean values of BP recorded at home. The number and doses of the blood lowering medications were noted.

After PTA, patients were stratified as those who achieved targets in SBP and DBP control according to guidelines, e.g., SBP less than 130 mmHg, and DBP below 80 mmHg. The prevalence of achieving the target SBP and DBP levels as well as the therapeutic failure were calculated.

RF assessment including levels of serum creatinine and eGFR were obtained prior to PTA. The eGFR was estimated from the Modification of Diet in Renal Disease (MDRD) formula, according to following equation: MDRD = 175 × creatinine [mg/dL] − 1.154 × age [years] − 0.203 × 0.742 [if female].

At baseline and after the PTA, patients were stratified into stages of chronic kidney disease (CKD) according to the Kidney Dialysis Outcomes Initiative (KDOQI) classification based on the MRDR formula for calculating the eGFR as follows: stage 1—eGFR above 90 mL/min/m^2^; stage 2—eGFR between 60–89 mL/min/m^2^; stage 3A—eGFR between 45–59 mL/min/m^2^; stage 3B—eGFR between 30–44 mL/min/m^2^ stage 4—eGFR between 15–29 mL/min/m^2^; and stage 5—eGFR less than 15 mL/min/m^2^ [17]. 

### 2.2. Biochemical Tests

All patients had fasting blood samples obtained on a patient admission to the department, prior to PTA procedure, as soon as the signed informed consent was obtained. Serum blood tests included glucose, glycated hemoglobin (HbA1C), high-sensitivity C-reactive protein (hs-CRP), creatinine, high-density-lipoprotein (HDL-C), low-density-lipoprotein (LDL-C) cholesterol and Triglycerides levels.

The well-controlled T2DM was defined as glycemic target goals achievement such as HbA1C < 7% (<53 mmol/mol) and mean fasting glucose concentration below <7.9 mmol/L [18].

### 2.3. Renal Doppler Ultrasonography (DUS)

The DUS was performed with the patient in a supine and the left or right lateral position, depending on which renal artery was assessed. Assessments were performed by 2 operators, using a high-resolution ultrasound machine (TOSHIBA APLIO with a convex probe). The following parameters were assessed: the systolic velocity in aorta, the peak-systolic and the end-diastolic velocity in the index renal artery, the renal-aortic-ratio, resistive index in the renal artery, the intra-renal resistive index and the pole-to-pole kidney length of the index and contralateral kidneys.

### 2.4. Renal Artery Stenting

The detailed PTA procedure was described previously [19]. In brief, inclusion criteria were: ARAS exceeding 60% lumen reduction on imaging studies (Doppler ultrasound or Computed tomography angiography (angio-CT), and confirmed on renal artery angiography. All patients received dual antiplatelet therapy before the procedure, which was continued for 3 months after PTA, afterwards single antiplatelet therapy was continued indefinitely. The choice of stent type and route of vascular access was left to the individual operator’s discretion. Prior to revascularization all patients were prepared with hyperhydration and temporary cessation of metformin.

### 2.5. Follow-Up and Reporting of Outcomes

The 24-months prospective follow-up was performed for renal and cardiovascular outcomes. The achieved values of the SBP, the DBP, the number of BP lowering medications, levels of serum creatinine and eGFR, progression to RRT, and glycemic control were recorded in all patients between 6 and 12 months, and at 24 months follow-up visits. Also, the status of achieved 2TDM control (well vs. ill-controlled diabetes) was assessed in all patients. During the follow-up period, patients were consulted by the diabetologist, who provided treatment modifications if required.

Subjects were categorized as those who achieved optimal SBP and DBP values vs. those who did not achieved target goals in SBP and DBP values. The optimal SBP was defined as values between 120 and 130 mmHg, and DBP as values between 70 and 80 mmHg, according to scientific recommendations for cardiovascular risk prevention in diabetic patients [13]. Parameters of achieved T2DM control were analyzed as a dichotomous variable (well vs. ill-controlled) according to contemporary recommendations, with a target HbA1C < 7.0% (<53 mmol/mol) and mean fasting glucose concentration below <7.9 mmol/L [18].

DUS was performed in all patients at follow-up visits. The incidence of recurrent renal artery stenosis was recorded. The diagnosis of the in-stent restenosis (ISR) on DUS was verified by the renal angiography with subsequent endovascular treatment when applicable. Patients who underwent ISR treatment were scheduled to 12-month follow-up for BP, RF and the outcomes.

The incidences of cardiovascular death, MI and stroke as well as composite end-point (MACCE) were recorded prospectively during a median follow-up period of 44 months (interquartile range; IQR: 23; 94). Adverse events were defined as fatal or non-fatal stroke, fatal or non-fatal MI, or cardiovascular death (i.e., any sudden or unexpected death unless proven as non-cardiovascular on autopsy). MI was diagnosed according to criteria of the European Society of Cardiology. Diagnosis of stroke was to be given by a neurologist to ensure reliability. RRT was classified as continuous, or temporary at the time of procedure (30 days periprocedurally, then censored). Final visit at closing data-base was done through telephone contact with a patient or appointed family member. There were no patients lost to follow-up.

### 2.6. Statistical Analysis

Continuous variables are presented as mean ± one standard deviation (SD) for variables with proven normal distribution by Shapiro-Wilk test, and median with interquartile range (IQR) for variables with no normal distribution. Categorical variables are expressed as frequencies and percentages (*n*, %). Means of analyzed parameters across groups were tested with the analysis of variance (ANOVA) test, and frequencies were compared by the chi-square test for independence. After PTA, achievements in SBP and DBP were analyzed as continuous variables, and as a dichotomous variable (optimal vs. non-optimal BP) in patients with adverse outcome vs. event-free. Parameters of achieved T2DM control were analyzed as a dichotomous variable (well vs. ill-controlled) according to contemporary recommendations. The potential independent prognostic markers of outcomes during the follow-up period were established from the clinical, biochemical and procedural variables with a Cox proportional hazard univariate analysis, and in case of significant difference (*p* < 0.05), they were entered into a multivariate Cox proportional hazard analysis model. The results of the uni- and multivariate Cox proportional hazard analysis were expressed as hazard ratio (HR) and 95% confidence interval (95%CI). The Kaplan-Meier event-free survival curves after renal artery stenting were constructed for independent parameters associated with outcomes. Statistical analyses were performed with Statistica 13.0 software. Statistical significance was assumed at *p*-value < 0.05.

## 3. Results

### 3.1. Basline Patients Characteristics

The mean age of the study participants was 69.4 ± 8.6 (range 47–84) years old. Forty-seven (50.5%) were females. Hypertension, hyperlipidemia, smoking, CAD, previous MI and stroke were diagnosed in 100%, 100%, 59.1%, 75.3%, 26.9%, and 17.2%, respectively. During 2 years preceding the PTA, a progressive CKD was documented in 22 (23.7%) patients. In thirteen patients, T2DM was diagnosed within the last 5 years, while in the remaining patients, T2DM diagnosis was set over 5 years. Twenty-nine patients were on insulin treatment, while 64 patients were on oral hypoglycemic medications. The detailed study group participants and the procedure characteristics is given in Table 1.

Altogether, 21 PTAs for bilateral or the single functional kidney and 72 PTAs for unilateral ARAS were performed, resulting in a significant reduction in the SBP (140.5 ± 21.6 vs. 159 ± 27.1 mmHg; *p* < 0.001), the DBP (75.5 ± 12.3 vs. 83.5 ± 12.7 mmHg; *p* < 0.001), the number of blood lowering medications (3.79 ± 1.32 vs. 4.24 ± 1.27; *p* < 0.001). There was a non-significant eGFR increase (52.0 ± 22.8 vs. 47.8 ± 21.2; *p* = 0.236), or serum creatinine reduction (*p* = 0.554) at 24-months follow-up, compared to the baseline values. 

Reduction of the doses, or the number of blood lowering medications were possible in 34 (37.8%) patients. None patient had a blood lowering agents discontinued. Six (6.5%) patients required intensification of a blood lowering treatment.

The proportions of patients achieving a SBP goal of <130 mmHg, a DBP goal of <80 mmHg and glycemic control were 41 (44.1%), 61 (65.6%), and 39 (41.9%) at 24-month follow-up. 

During median follow-up period of 44 months (IQR, 23; 94), MACCE occurred in 41 (44.1%) patients, including 17 (18.3%) cardiovascular deaths, 18 (19.4%) non-fatal MIs, and 11 (11.8%) non-fatal ischemic strokes. Progression of CKD reaching end-stage renal disease (ESRD) requiring long-life RRT occurred in 6 (6.5%) patients, while 4 (4.3%) patients went on the peri-procedural short time RRT.

Overall, MACCE-RRT occurred in 46 (49.5%) patients, including 5 patients having both MACCE and RRT endpoints. Comparison of patients with and without a composite endpoint depending on the baseline patients’ characteristic is given in Table 1. In brief, patients with MAC-CE-RRT, compared to event-free group did not differ with regard to age, body mass index, previous MI and stroke, prevalence of CAD and peripheral arterial disease, type of T2DM treatment, baseline stage of CKD, or baseline values of SBP, DBP, LDL-C, HDL-C, triglycerides, hs-CRP, and serum creatinine, DUS parameters, angiographic ARAS severity, and implanted stent diameter, or its length (Table 1). Only, men were more prevalent in MACCE-RRT group.

### 3.2. Renal and Cardiovascular Outcomes and Follow-Up Blood Pressure, Renal Function and Glycemic Control Parameters

In patients with a combined outcome, the proportion of patients achieving target goal for T2DM control was lower as compared to event-free group (12/46; 26.1% vs. 27/47; 57.4%; *p* = 0.002), and it was regardless of T2DM treatment type, Table 2.

Optimal values of SBP were observed in 26 (55.3%) of 47 patients with event-free survival, compared to 15 (32.6%) of 46 with MACCE-RRT (*p* = 0.027), while optimal DBP in 37 (78.7%) and 24 (52.2%) patients (*p* = 0.007), respectively, Table 2. Also, in event-free patients, final SBP and DBP values were significantly lower, as compared to MACCE-RRT group (SBP: 136.7 ± 15.8 vs. 147.8 ± 25.8 mmHg, *p* = 0.006; and DBP: 74.4 ± 12.3 vs. 80.8 ± 13.3 mmHg, *p* = 0.009), while eGFR was non-significantly higher (54.4 ± 19.2 vs. 47.2 ± 25.4, *p* = 0.172). Instead, there was higher incidence of eGFR values below 45 mL/min/m^2^ in patients with MACCE-RRT vs. event-free patients (28/46; 60.9%, vs. 16/47; 34%; *p* = 0.029), and respectively higher incidence of ISR (39.1% vs. 12.8%; *p* = 0.004), Table 2.

### 3.3. Univariate and Multivariate Cox Proportional Hazard Analysis

Univariate Cox proportional hazard analysis indicated several parameters that may have an impact on the increased risk of MACCE-RRT including post procedural CKD in stages 3B to 5, compared to 1–3A (eGFR < 45 mL/min/m^2^ vs. eGFR ≥ 45 mL/min/m^2^) (HR, 2.77; 95% CI, 1.51 to 5.07; *p* < 0.001), peripheral arterial disease (HR, 1.98; 95% CI, 1.11 to 3.58, *p* = 0.021), previous stroke (HR, 2.11; 95% CI, 1.02 to 4.37, *p* = 0.043), and baseline renal-aortic-ratio for index ARAS (HR, 1.32; 95% CI, 1.12 to 1.57, *p* < 0.001) (Table 3). While, female gender (HR, 0.46, 95% CI 0.25 to 0.87, *p* = 0.016) and well-maintained target goal for T2DM control at 24-months F-U (HR, 0.27; 95% CI, 0.13 to 0.55, *p* < 0.001), but not an optimal glycemic target before the PTA (HR, 0.62; 95%CI, 0.34–1.14, *p* = 0.126) were associated with reduced risk for outcomes. There was also trend to associations for hs-CRP, baseline SBP and prior hypertension crisis (Table 3).

Among these, on multivariate Cox analysis, only achieved glycemic target of T2DM (HR, 0.27, 95% CI, 0.13–0.57, *p* < 0.001), the eGFR below 45 mL/min/m^2^ (HR, 2.20; 95%CI, 1.20–4.04; *p* = 0.011), and previous stroke (HR, 2.52; 95%CI, 1.19 to 5.34, *p* = 0.015) retained their associations with MACCE-RRT. The detailed parameters of univariate and multivariate Cox hazard analysis are shown in Table 3.

### 3.4. Kaplan-Meier Event Free Survival

**Figure 2:**
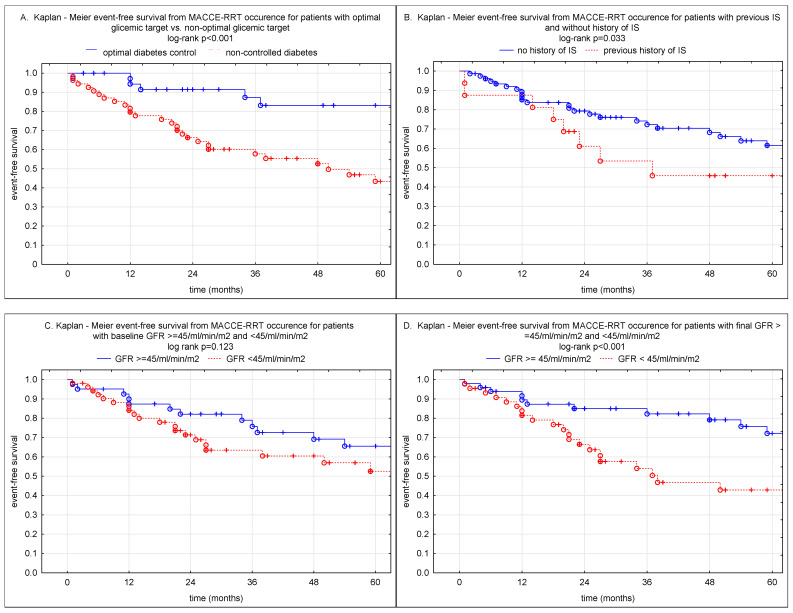

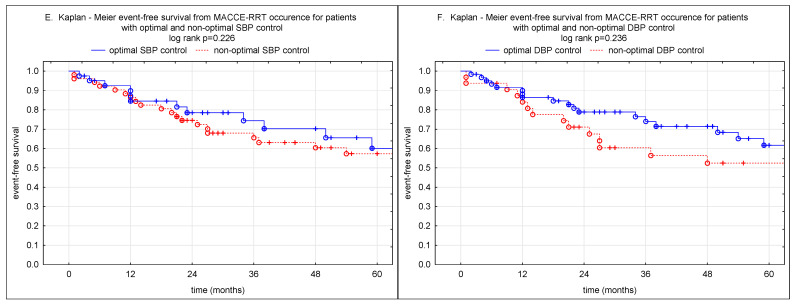
The Kaplan-Meier plot depicts survival after renal artery stenting for parameters associated with outcome. (**A**) patients with maintained glycemic target goals (continuous blue line) and patients with non-optimal diabetic control (discontinuous red line). (**B**) patients with previous stroke (discontinuous red line) and non-stroke (continuous blue line). (**C**) patients with baseline eGFR ≥ 45 mL/min/m^2^ (continuous blue line) and the eGFR < 45 mL/min/m^2^ (discontinuous red line). (**D**) patients with final, post intervention, eGFR ≥ 45 mL/min/m^2^ (continuous blue line) and the eGFR < 45 mL/min/m^2^ (discontinuous red line). (**E**) patients with optimal target goals of SBP after PTA (continuous blue line) and patients with non-optimal SBP control (discontinuous red line). (**F**) patients with optimal target goals of DBP after PTA (continuous blue line) and patients with non-optimal DBP control (red discontinuous line). Ticks along the lines represent censored cases. The log-rank test *p*-value between the two groups is presented in each figure.

At 3-years patient free-survival rates from MACCE-RRT were 86% vs. 58% in whose who had maintained glycemic goal versus non-optimally controlled T2DM (log-rank *p* < 0.001); 85% vs. 54% in patients with a final eGFR above 45 mL/min/m^2^ vs. lower eGFR values (log-rank *p* < 0.001); 72% vs. 45% for whose without stroke history vs. former stroke (log-rank 0.033); 75% vs. 66% for optimal vs. non-optimal targets goals in SBP (log-rank *p* = 0.226), and 74% vs. 60% for optimal vs. non-optimal DBP (log-rank *p* = 0.236), and 76% vs. 63% in patients with pre-intervention eGFR > 45 vs. <45 mL/min/m^2^ (Figure 2).

## 4. Discussion

The majority of randomized clinical trials could not confirm the impact of the intervention on ARAS on the improvement of hypertension control, RF, cardiovascular events, and mortality [20]. Nowadays, according to guidelines, a first choice management in the treatment of ARAS is an optimal medical therapy, including statins, antihypertensive agents, platelet inhibition, and intensive cardiovascular risk factors control [11,12].

Conversely, presence of severe ARAS is a strong risk factor of the cardiovascular and all-cause mortality [6,7]. Furthermore, results of the clinical series showed that revascularization of the stenotic lesion compared with medical therapy may be potentially associated with renal and cardiovascular prognosis improvement [10,21,22]. Thus, data gathered in this field strongly suggest individualized and personalized attitude as they are mostly ambiguous [23,24,25].

Therefore, the selection of subgroups and appropriate indications for better outcomes is necessary. A very specific subgroup of patients constitute individuals with T2DM, where the mechanisms of both kidney damage and hypertension are complex and multi-factorial [4].

It is debatable whether diabetes may influence results of renal artery stenting in patients with poorly controlled hypertension (despite multi-pill blood lowering strategy) in the setting of the concomitant severe ARAS. 

In our study, MACCE-RRT was observed in 49.5% of diabetic patients at follow-up, compared to general population where adverse outcomes were reported in 30% to 75% of patients undergoing PTA for ARAS [19,22,23,26]. However, meta-analysis of randomized and non-randomized studies showed the effect size of 0.55 to 2.35 for all-cause death and cardiovascular mortality, with no statistically significant differences between PTA and medical therapy alone [27].

Hu et al. indicated in a group of 230 patients followed for 36 months, that T2DM (OR, 2.15; 95%CI 1.1 to 4.1) is independently associated with risk of renal and cardiovascular adverse events after renal artery stent implantation for ARAS, together with age ≥ 65 years old, Charlson comorbidity index score of ≥2 points, previous stroke and congestive heart failure [23]. Similarly, higher all-cause mortality rates were demonstrated among 138 patients with diabetes (HR 1.42; 95% CI, 1.09 to 1.86) out of the total 398 patients enrolled in Takahashi et al. study [7].

On the contrary, in Dregoesc et al. study outcomes were independent of diabetes mellitus [26].

In our present study, retrospectively analyzing the outcome of endovascular treat-ment for ARAS in diabetic patients, we showed varied results. We observed that post pro-cedural glycemic control, CKD stage 3B to 5, and previous stroke are independently relat-ed to the incidence of MACCE-RRT.

In diabetics poor RF, before and after operation, was associated with progression to dialysis and death [28]. In our study, the CKD in stages from 3B to 5 were associated with a 2.2-fold (95% CI, 1.2 to 4.04) risk increase of MACCE-RRT after PTA for ARAS. In line, in the study of Dregoesc et al., including 65 patients, the post PTA CKD in classes 3B to 5 were associated with a 5.8-fold (95% CI, 1.5–27.9; *p* = 0.01) risk increase of the long-term mortality, along with age, male gender and uncontrolled hypertension [26]. Similarly, in Misra et al. study, in multivariable analysis, high-grade proteinuria and CKD in stages 3B to 5 vs. 1 to 3A were independently associated with increased risk of progression to RRT, and all-cause mortality [6]. While, angiotensin-converting enzyme inhibitor/angiotensin receptor blocker use was associated with decreased risk of progression to RRT, and statins use with decreased risk of all-cause mortality [6].

Importantly, Takahashi et al. clearly showed that post-intervention eGFR improvement for every 1 unit increase in eGFR up to 40 mL/min/1.73 m^2^ among patients with post-interventional eGFR < 40 mL/min/1.73 m^2^ was associated with decreased RRT and mortality risk (HR 0.95; 95% CI, 0.94–0.97; *p* < 0.001) [7]. These findings suggest that patients with advanced CKD and ARAS may benefit from revascularization especially when their pre-intervention eGFR is lower than 40/mL/min/1.73 m^2^.

In our present study, a mean eGFR increase at follow-up was rather modest, yet, 7 patients with initially eGFR below 45 mL/min/m^2^ had increased their eGFR value above 45 mL/min/m^2^ at FU. This modest improvement in RF occurred highly prognostic for MACCE-RRT incidence reduction and it was associated with favorable outcomes (log rank *p* < 0.001).

Previous clinical series indicated improved RF after intervention on ARAS to be associated with improved survival; however, it was difficult to predict who will respond to the treatment [29,30,31,32]. 

In our former study, we have demonstrated that improvement in RF parameters can be expected when initial pre interventional creatinine level exceeds 122 µmol/L, but eGFR is above 30 mL/min/1.73 m^2^, the index kidney length above 98 mm, renal artery end-diastolic velocity above 1.1 m/s, and arterial resistive index is below 0.74 [19]. Furthermore, the increase in the eGFR of at least 11 mL/min/1.73 m^2^ was independently associated with a reduced risk of death (HR, 0.42; 95% CI, 0.19–0.90; *p* = 0.02) and MACCEs (HR, 0.54; 95% CI, 0.32–0.93; *p* = 0.03), while a decrease of DBP by 5 mm Hg or higher, with a reduced risk of stroke (HR, 0.1; 95% CI, 0.02–0.39; *p* = 0.001) at 12-month follow-up [32]. This is particularly important as eGFR is an independent predictor of cardiovascular and renal outcomes in subjects with T2DM [33,34].

The novelty of our present study was the attempt to assess whether the maintenance of the glycemic target goals (a mean fasting glucose < 7.9 mmol/L and HbgA1C < 7%) at FU, may have an impact on the outcome in patients treated with PTA for ARAS. We have evidenced that the well-maintained glycemic control was independently associated with a 73% (95% CI, 0.13 to 0.57) risk reduction in the incidence of MACCE-RRT.

Of note, the preoperative glycemic control was not associated with postoperative MACCE-RRT. Thus, it shows that the post procedural well maintained glycemic control is vital for a favorable renal and cardiovascular outcome.

To our knowledge, this is the first study to address the relationship between post intervention glycemic control and the renal and cardiovascular outcomes. One can only speculate whether obtained and maintained glycemic targets would be associated with better outcomes also in diabetic patients on the optimal medical therapy alone, but without the intervention on the ARAS.

The relationship between previous stroke and the outcome of renal artery stenting is difficult to explain by the fact of PTA. This finding was also observed by Hu et al., however worse cardiovascular prognosis after stroke may result from the general morbidity burden in this subgroup of patients. It should be remembered that atherosclerosis is a progressive disease inevitably leading to new adverse events, and patients with ARAS are particularly at risk of multivessel athero-occlusive disease [35,36,37].

Many trials and observational studies of diabetes and hypertension treatment clearly demonstrated that long-term poorly controlled hypertension damages the kidneys, and both BP and the duration of hypertension are correlated with the risk of CKD and ESRD [38]. In patients with ARAS, invariably associated with systemic atherosclerosis, atherosclerotic risk factors must be controlled, with pharmacotherapy focused on target-level driven control of BP, and glycemic and lipid levels [39].

Importantly, in observational studies, most of patients, including the diabetic, have a beneficial blood BP response after intervention on ARAS [19,23,26,31,32,40]. In fact, significant BP reduction following PTA for ARAS is commonly reported from clinical series [19,23,26,31,32,40,41]. We have observed significant reduction in the post intervention mean SBP and DBP values, as well as in the number of blood lowering medications, compared to the pre-intervention values. The mean SBP and DBP decrease were 19 mmHg for SBP and 8 mmHg for DBP at final FU, respectively (both *p* < 0.05). Additionally, patients who suffered from MACCE-RRT did not differ with respect to initial pre-intervention BP values. While, at follow up, both mean SBP and DBP were significantly higher in those patients who had composite outcome as compared to event free patients. However, neither BP values at FU, nor higher prevalence of patients who obtained optimal target goals in event-free group, as compared to lower prevalence in the outcome group, did not reach significance in Kaplan-Meier free-survival analysis, and Cox proportional hazard models. On the contrary, in general population with renovascular disease, a 5 mmHg or higher decrease in DBP pressure after PTA for ARAS was associated with reduction in stroke rate [19]. While, the study of Dregoesc et al., in post revascularization uncontrolled hypertension (OR 8.9; 95% CI 1.7–63.5; *p* = 0.01) was associated with long-term mortality [26].

Regretfully, association between BP change after PTA for ARAS and adverse outcomes was rather not observed in either randomized studies and clinical series [5,9,25,26,27,28,29]. Thus, analyzing the available experience from previously published studies, RF change following PTA for ARAS seems more important for the outcomes, than change in the BP values [22,23,41,42]. Additionally, further studies are needed to investigate role of novel biomarkers for assessment of the renal and cardiovascular outcomes after revascularization of ARAS. One of the promising directions is assessment of the activation of mesenchymal stem cells and their role in the renal tissue regeneration after kidney reperfusion leading to repair organ damage [43]. 

## 5. Study Limitations

Our study has some limitations. We did not assess the impact of diabetes treatment modifications during the follow-up period, which could have the potential impact on the renal and cardiovascular outcomes. Nevertheless, we used the achievement of the goals in the glycemic control as the point of reference for the risk assessment.

## 6. Conclusions

In conclusion, in diabetic patients who underwent PTA for ARAS there is significant reduction in blood pressure values, however with no impact on the outcomes. Renal function with a GFR above 45 mL/min/1.73 m^2^ and optimal diabetes control are independent predictors of outcome.

## Figures and Tables

**Table 1:** Baseline characteristics of 93 study participants with atherosclerotic renal artery stenosis according to clinical, renal Doppler ultrasonography and angiographic status.

Variable	All Study Participants N = 93	MACCE-RRT (−) N = 47	MACCE-RRT (+) N = 46	*p*-Value
Demographic data				
Age, y, mean (SD)	69.3 (7.2)	70.2 (9.1)	68.3 (8.2)	0.136
Female, *n* (%)	47 (50.5)	30 (63.8)	17 (37)	0.009
Hypertension, *n* (%)	93 (100)	47 (100)	46 (100)	n/a
Systolic blood pressure, mmHg, mean (SD)	159 (27.1)	158.0 (24.1)	159.8 (30.6)	0.382
Diastolic blood pressure, mmHg, mean (SD)	83.5 (12.7)	83.3 (12.2)	84.2 (14.0)	0.371
Number of blood lowering medications, mean (SD)	4.22 (1.26)	4.40 (1.3)	4.04 (1.17)	0.097
Previous hypertension crysis, *n* (%)	36 (38.7)	18 (38.3)	18 (39.1)	0.867
Previous pulmonary flash oedema, *n* (%)	9 (9.7)	4 (8.5)	5 (10.9)	0.751
Diabetes, *n* (%)	93 (100)	47 (100)	46 (100)	n/a
Insulin, *n* (%)	29 (31.2)	15 (31.9)	14 (30.4)	0.887
Sulfonylurea, *n* (%)	23 (24.7)	13 (27.7)	10 (21.7)	0.768
Metformin, *n* (%)	37 (39.8)	17 (36.2)	20 (43.4)	0.472
GLP-1 receptor agonists, *n* (%)	1 (1.1)	1 (2.1)	0 (0)	-
SGLT2 inhibitors, *n* (%)	3 (3.2)	1 (2.1)	2 (4.3)	0.545
DPP4 inhibitors, *n* (%)	0 (0)	0 (0)	0 (0)	-
Fasting glucose (mmol/L), mean (SD)	7.25 (2.66)	6.98 (2.93)	7.57 (2.47)	0.301
HBA1c (%), mean (SD)	7.12 (1.99)	7.09 (1.93)	7.14 (2.07)	0.098
Renal function before PTA procedure				
Documented renal function deterioration, *n* (%)	22 (23.7)	12 (25.5)	10 (21.7)	0.630
Baseline serum creatinine level, µmol/L, mean (SD)	135.5 (55.6)	131.7 (46.5)	141.3 (58.8)	0.203
stage 1 (eGFR > 90 mL/min/m^2^), *n* (%)	6 (6.5)	2 (4.3)	4 (8.7)	-
stage 2 (eGFR: 60–89 mL/min/m^2^), *n* (%)	18 (19.4)	7 (14.9)	11 (23.9)	-
stage 3A (eGFR: 45–59 mL/min/m^2^), *n* (%)	18 (19.4)	13 (27.7)	5 (10.9)	0.219 *
stage 3B (eGFR: 30–44 mL/min/m^2^)	34 (36.6)	18 (38.3)	16 (34.8)	-
stage 4 (eGFR: 15–29 mL/min/m^2^), *n* (%)	16 (17.2)	7 (14.9)	9 (19.6)	-
stage 5 (eGFR < 15 mL/min/m^2^), *n* (%)	1 (1.1)	0 (0)	1 (2.2)	-
Smoking (past or current), *n* (%)	55 (59.1)	25 (53.2)	30 (65.2)	0.229
Co-existing coronary artery disease (lesions >50%), *n* (%)	70 (75.3)	34 (72.3)	36 (78.2)	0.764
Previous myocardial infarction, *n* (%)	25 (26.9)	14 (29.8)	11 (23.9)	0.766
Left ventricular ejection fraction, %, mean (SD)	56.5 (11.3)	57 (11.3)	56 (11.2)	0.364
Body mass index (kg/m^2^), mean (SD)	29.5 (4.5)	29 (4.1)	30.1 (4.1)	0.249
Previous ischemic stroke, *n* (%)	16 (17.2)	10 (21.3)	6 (13)	0.293
Peripheral arterial disease, *n* (%)	43 (46.2)	19 (40.4)	24 (52.2)	0.256
Dyslipidemia, *n* (%)	93 (100)	47 (100)	46 (100)	n/a
Total cholesterol, mmol/L, mean (SD)	4.86 (1.36)	4.43 (1.13)	5.21 (1.44)	0.329
LDL-C, mmol/L, mean (SD)	2.77 (1.18)	2.50 (0.96)	3.02 (1.32)	0.184
HDL-C, mmol/L, mean (SD)	1.23 (0.35)	1.23 (0.33)	1.23 (0.39)	0.473
Triglycerides, mmol/L, mean (SD)	2.22 (1.95)	2.33 (2.48)	2.11 (1.21)	0.498
Hs-CRP, mg/dL, mean (SD)	5.1 (5.43)	3.81 (3.07)	6.49 (7.05)	0.375
Selected procedural data during PTA for ARAS				
Renal artery lumen stenosis, %, mean (SD)	78.4 (13.5)	79.5 (13.8)	77.2 (13.2)	0.197
PTA of unilateral ARAS, %, mean (SD)	72 (77.4)	40 (85.1)	32 (69.6)	
PTA of bilateral ARAS, %, mean (SD)	17 (18.3)	7 (14.9)	10 (21.7)	0.073 **
PTA of single functional kidney, %, mean (SD)	4 (4.3)	0 (0)	4 (8.7)	
Stent implantation (per patient), %, mean (SD)	93 (100)	47 (100)	46 (100)	n/a
Stent diameter, mm, mean (SD)	5.65 (0.93)	5.67 (0.82)	5.72 (0.96)	0.483
Stent length, mm, mean (SD)	16.3 (5.7)	15.8 (4.9)	17.4 (6.9)	0.215
Renal doppler ultrasonography parameters before PTA				
PSV in index renal artery, m/s, mean (SD)	3.73 (1.1)	3.63 (1.22)	3.83 (0.94)	0.086
EDV in index renal artery, m/s, mean (SD)	0.93 (0.4)	0.83 (0.33)	1.02 (0.44)	0.467
Renal-aortic-ratio for index renal artery, mean (SD)	4.5 (1.54)	4.0 (1.22)	5.0 (1.68)	0.054
Resistive index in the index renal artery, mean (SD)	0.75 (0.05)	0.76 (0.05)	0.75 (0.05)	0.229
Intrarenal resistive index in the index kidney, mean (SD)	0.66 (0.09)	0.67 (0.08)	0.65 (0.09)	0.498
Index kidney length (mm), mean (SD)	100.4(11.2)	100.3 (9.2)	100.5 (13)	0.460
Contralateral kidney length (mm), mean (SD)	103.5(18.9)	106.8(17.2)	100.3 (20.1)	0.063

ARAS: atherosclerotic renal artery stenosis; DPP4: dipeptidyl peptidase 4; EDV: end diastolic velocity; eGFR: estimated glomerular filtration rate; GLP-1: glucagon-like peptide-1; HDL-C: high density lipoprotein cholesterol; hs-CRP: high sensitivity C-Reactive Protein; LDL-C: low density lipoprotein cholesterol; PTA: percutaneous transluminal angioplasty; PSV: peak systolic velocity; SD: standard deviation; SGLT2 inhibitor: sodium glucose cotransporter 2 inhibitor. * *p*-value between renal stage below 45 mL/min/m^2^ vs. 45 mL/min/m^2^ and higher. ** *p*-value for unilateral ARAS vs. bilateral and single functional kidney.

**Table 2:** Final follow-up post-procedural parameters of blood pressure, renal function, blood lowering medications, glycaemic and biochemical tests, and stent patency in MACCE-RRT (+) vs. MACCE-RRT (−) patients.

Final Follow-Up Post-Procedural Parameters	MACCE-RRT (−) N = 47	MACCE-RRT (+) N = 46	*p*-Value
Fasting glucose (mmol/L), mean (SD)	7.13 (3.25)	8.10 (3.72)	0.140
HbA1C (%), mean (SD)	6.3 (2.2)	7.8 (2.8)	0.010
Maintained target goal for T2DM control, *n* (%)	27 (57.4)	12 (26.1)	0.002
LDL-cholesterol (mmol/L), mean (SD)	2.48 (0.86)	2.77 (1.17)	0.112
Systolic blood pressure, mmHg, mean (SD)	136.7 (15.8)	147.8 (25.8)	0.006
Diastolic blood pressure, mmHg, mean (SD)	74.4 (12.3)	80.8 (13.3)	0.009
Optimal SBP target goal (120–130 mmHg)	26 (55.3)	15 (32.6)	0.027
Optimal DBP target goal (70–80 mmHg)	37 (78.7)	24 (52.2)	0.007
Number of blood lowering medications, mean (SD)	3.70 (1.2)	3.87 (1.47)	0.273
Serum creatinine level, µmol/L, mean (SD)	117.3 (51.9)	150.1 (69.4)	0.006
eGFR, mL/min/m^2^, mean (SD)	54.4 (19.2)	47.2 (25.4)	0.172
eGFR < 45 mL/min/m^2^, *n* (%)	16 (34)	28 (60.9)	0.029
stage 1 (eGFR > 90 mL/min/m^2^), *n* (%)	3 (4.3)	5 (10.9)	-
stage 2 (eGFR: 60–89 mL/min/m^2^), *n* (%)	15 (31.9)	5 (10.9)	-
stage 3A (eGFR: 45–59 mL/min/m^2^), *n* (%)	13 (27.7)	8 (17.4)	-
stage 3B (eGFR: 30–44 mL/min/m^2^)	13 (27.7)	16 (34.8)	-
stage 4 (eGFR: 15–29 mL/min/m^2^), *n* (%)	3 (6.3)	7 (15.2)	-
stage 5 (eGFR < 15 mL/min/m^2^), *n* (%)	0 (0)	5 (10.9)	-
In-stent restenosis of index lesion, *n* (%)	6 (12.8)	18 (39.1)	0.004
Renal replacement therapy, *n* (%)	-	10 (21.7) *	-
Transient (peri-procedural) RRT, *n* (%)	-	4	-
Permanent RRT, *n* (%)	-	6	-
Major adverse cardiac and cerebral event, *n* (%)	-	41 (89.1) *	-
Cardiovascular death, *n* (%)	-	17 (37) *	-
Non-fatal stroke, *n* (%)	-	11 (23.9) *	-
Non-fatal myocardial infarction, *n* (%)	-	18 (39.1) *	-

* some patients had both major cardiac and cerebral event and renal replacement therapy.

**Table 3:** Univariate and multivariate Cox proportional hazard analysis for the incidence of major cardiac and cerebral events and renal replacement therapy following endovascular treatment for atherosclerotic renal artery stenosis.

	Univariate Cox Proportional Hazard Analysis			Multivariate Cox Proportional Hazard Analysis		
Clinical Parameter	Hazard Ratio	95% Confidence Interval	*p*-Value	Hazard Ratio	95% Confidence Interval	*p*-Value
Pre procedural and angiographic data						
Age	1.02	0.98–1.06	0.200			
Female gender	0.46	0.25–0.87	0.016	0.75	0.36–1.56	0.466
Smoking	1.32	0.71–2.48	0.371			
Body Mass Index	0.95	0.88–1.03	0.235			
Coronary artery disease	0.81	0.41–1.57	0.530			
Multivessel coronary artery disease	1.26	0.71–2.26	0.429			
Previous myocardial infarction	1.77	0.93–3.36	0.081			
Left ventricle ejection fraction	0.99	0.96–1.02	0.633			
Peripheral arterial disease	1.98	1.11–3.58	0.021	1.73	0.85–3.53	0.130
Previous stroke	2.11	1.02–4.37	0.043	2.52	1.19–5.34	0.015
Achieved glycemic target before PTA	0.62	0.34–1.14	0.126			
Baseline fasting glucose	1.05	0.92–1.21	0.449			
Insulin treatment	1.61	0.84–3.10	0.147			
Baseline creatinine	1.00	0.99–1.01	0.073	1.00	0.99–1.01	0.473
Baseline eGFR	0.99	0.98–1.01	0.327			
Baseline hs-CRP	1.04	0.99–1.08	0.055	0.98	0.94–1.04	0.687
Baseline LDL-cholesterol	0.98	0.99–1.00	0.524			
Baseline systolic blood pressure	1.01	0.99–1.02	0.072	1.00	0.98–1.02	0.625
Baseline diastolic blood pressure	1.01	0.98–1.03	0.536			
Prior pulmonary flash oedema	1.18	0.41–3.35	0.753			
Prior hypertensive crisis	1.80	0.96–3.36	0.064	1.13	0.38–3.37	0.817
Baseline CKD (eGFR < 45 mL/min/m^2^)	0.62	0.34–1.14	0.124			
Bilateral/single kidney vs. unilateral PTA	1.53	0.54–4.34	0.419			
Degree of renal artery stenosis	1.01	0.98–1.04	0.336			
Stent diameter	1.00	0.73–1.38	0.975			
Stent length	1.00	0.95–1.05	0.909			
RAR in index RAS	1.32	1.12–1.57	< 0.001	1.20	0.99–1.45	0.059
RI in index RAS	1.71	0.01–2041	0.880			
IRI in index RAS	1.44	0.04–54.9	0.842			
Post procedural data						
Achieved target glycaemic goals	0.27	0.13–0.55	<0.001	0.27	0.13–0.57	<0.001
CKD (eGFR < 45 mL/min/m^2^)	2.77	1.51–5.07	<0.001	2.20	1.20–4.04	0.011
Change in creatinine level	1.00	0.99–1.01	0.128			
Follow-up eGFR	0.98	0.96–0.99	0.017			
Change in the eGFR	0.99	0.97–1.00	0.141			
Follow-up systolic blood pressure	1.00	0.99–1.02	0.302			
Change in systolic blood pressure	0.99	0.98–1.01	0.283			
Obtained treatment goal for SBP	0.68	0.37–1.27	0.233			
Follow-up diastolic blood pressure	1.01	0.99–1.03	0.306			
Change in diastolic blood pressure	1.01	0.98–1.03	0.636			
Obtained treatment goal for DBP	0.71	0.39–1.27	0.253			
In-stent restenosis	1.51	0.81–2.81	0.194			

## Data Availability

The data presented in this study are available on request from the corresponding author. The data are not publicly available due to privacy.

## Abbreviations

ARAS	atherosclerotic renal artery stenosis
BP	blood pressure
CKD	chronic kidney disease
CAD	coronary artery disease
CVD	cardiovascular disease
DBP	diastolic blood pressure
DUS	renal ultrasonography
ESRD	end-stage renal disease
eGFR	estimated glomerular filtration rate
HbA1C	glycated hemoglobin
HDL-C	high-density-lipoprotein cholesterol
hs-CRP	high-sensitivity C-reactive protein
MACCE	major cardiac and cerebral events
MI	myocardial infarction
LDL-C	low-density lipoprotein cholesterol
LVEF	left ventricle ejection fraction
PTA	stent-supported angioplasty
RF	renal function
RRT	renal replacement therapy
SBP	systolic blood pressure
T2DM	type 2 diabetes mellitus
